# Identification of *Streptococcus pyogenes* isolates with reduced beta-lactam susceptibility in a cohort of children with pharyngitis

**DOI:** 10.1128/aac.00354-26

**Published:** 2026-05-29

**Authors:** Daniel B. Amusin, Lacy M. Simons, Dulce S. Garcia, Samuel W. M. Gatesy, Sophia H. Nozick, Jennifer Sheridan, Alan R. Hauser, Emma Office, Preeti Jaggi, David B. Williams, Rachel J. Tanz, Yiannis Katsogridakis, Larry K. Kociolek, Ami B. Patel, Stanford T. Shulman, Judith A. Guzman-Cottrill, Robert R. Tanz, Egon A. Ozer

**Affiliations:** 1 Department of Microbiology-Immunology, Northwestern University Feinberg School of Medicine 12244, Chicago, Illinois, USA; 2 Department of Medicine, Division of Infectious Diseases, Northwestern University Feinberg School of Medicine 12244, Chicago, Illinois, USA; 3 Department of Pediatrics, Division of Infectious Diseases, Emory University School of Medicine 12239, Atlanta, Georgia, USA; 4 Stanley Manne Children’s Research Institute, Ann & Robert H. Lurie Children’s Hospital of Chicago, Chicago, Illinois, USA; 5 Desert Valley Pediatrics, Phoenix, Arizona, USA; 6 Department of Pediatrics, Northwestern University Feinberg School of Medicine 12244, Chicago, Illinois, USA; 7 Division of Pediatric Emergency Medicine, Ann & Robert H. Lurie Children’s Hospital of Chicago 2429, Chicago, Illinois, USA; 8 Division of Pediatric Infectious Diseases, Ann & Robert H. Lurie Children’s Hospital of Chicago 2429, Chicago, Illinois, USA; 9 Department of Pediatrics, Division of Infectious Diseases, Oregon Health and Science University Doernbecher Children’s Hospital 20240, Portland, Oregon, USA; 10 Division of Advanced General Pediatrics and Primary Care, Ann & Robert H. Lurie Children’s Hospital of Chicago 2429, Chicago, Illinois, USA; The Peter Doherty Institute for Infection and Immunity, Melbourne, Victoria, Australia

**Keywords:** penicillin, beta-lactam, pharyngitis, penicillin-binding protein, *Streptococcus pyogenes*

## Abstract

We investigated sequences of the penicillin-binding protein 2x (PBP2x) among 902 *Streptococcus pyogenes* isolates from children with pharyngitis. Twenty-five pharyngeal isolates carried variants associated with reduced β-lactam susceptibility. Penicillin and ampicillin susceptibility testing confirmed reduced susceptibility among PBP2x variant isolates relative to those with the wild-type sequence.

## INTRODUCTION

*Streptococcus pyogenes* (group A streptococcus [GAS]) is the most common cause of acute bacterial pharyngitis in both children and adults (1). Timely and appropriate antibiotic treatment of this infection is important for prompt resolution of symptoms, preventing transmission, and preventing acute rheumatic fever. β-Lactam antibiotics such as penicillin remain a mainstay of GAS treatment due to universal clinical susceptibility (2).

To date, no confirmed cases of clinical β-lactam resistance in GAS have been reported. However, Vannice et al. reported two isolates collected from invasive infections in Washington State in 2017 and 2018 with elevated minimum inhibitory concentrations (MICs) to ampicillin and amoxicillin. Both exhibited MICs at the breakpoint for resistance to ampicillin and contained missense variant T553K within the transpeptidase domain of penicillin-binding protein 2x (PBP2x) (3). Further studies of PBP2x and other penicillin-binding proteins, mostly in invasive GAS isolates, identified additional missense variants associated with reduced β-lactam susceptibility (RBLS) (4–8).

Although pharyngitis is the most common infection caused by GAS, the prevalence and impact of PBP2x variants in these infections remain uncharacterized in the United States (2, 8). To address this gap, we examined the PBP2x sequence of 902 pharyngeal isolates collected from pediatric patients with symptomatic pharyngitis in four cities within the United States (Chicago, IL; Portland, OR; Atlanta, GA; Phoenix, AZ) from 2020 to 2023 (Lurie Children’s Hospital Institutional Review Board #2019-2852). Swabs from patients positive by point-of-care antigen testing were cultured on tryptic soy agar with 5% sheep’s blood at 35°C for up to 48 h. Beta-hemolytic, catalase-negative colonies were identified as GAS by the PathoDX latex agglutination test. DNA was extracted using the QIAamp DNA Mini Kit (QIAGEN) after incubation in proteinase K (New England Biolabs), and whole-genome sequencing (WGS) libraries were constructed using the SeqWell ExpressPlex 2.0 kit. WGS was performed on an Illumina MiSeq instrument using the V3 kit to generate 300 bp paired-end reads.

Sequencing reads were trimmed with fastp v0.24.0 using default parameters and *de novo* assembled using SPAdes v4.0.0 with “automatic coverage cutoff” and “careful” settings (9, 10). Assembly contigs with average read depths of less than five or shorter than 200 bp were removed. Assembly quality was evaluated with CheckM v1.2.3, and only assemblies with greater than 95% completeness and less than 5% contamination were included (11). Isolate *emm* types and PBP2x variants within the transpeptidase domain were identified using the Centers for Disease Control and Prevention (CDC) *Streptococcus* laboratory bioinformatic analysis pipeline (https://github.com/BenJamesMetcalf/GAS_Scripts_Reference) (12). For isolates that failed PBP2x variant identification by this pipeline, the *pbp2x* gene transpeptidase-domain encoding sequence was identified by *in silico* PCR using SPIDER (https://github.com/RunningMSN/SPIDER), and variant types were assigned from the CDC pipeline sequence database.

The PBP2x sequence was identified in all 902 assemblies. Ten PBP2x variants were represented, each of which was previously cataloged in the CDC *Streptococcus* laboratory database (4). The most common alleles were wild-type (WT, 73.4%), G600D (12.0%), S562T (6.4%), and I502V, P676S (5.1%), comprising 96.9% of isolates. Four alleles previously associated with RBLS were identified in 25 isolates (4–6, 8). These variants and *emm* types of associated isolates included 17 M593T (12 *emm75* and 5 *emm4*); 6 P601L (4 *emm1*, 1 *emm11*, and 1 *emm87*); 1 P601H (*emm11*); and 1 S562T, M593T, P676S (*emm73*). Isolates with these variants had been collected in Chicago (13 M593T; 3 P601L; and 1 S562T, M593T, P676S) and Atlanta (4 M593T, 3 P601L, and 1 P601H). While an isogenic G600D mutant in an *emm*89 strain was reported to induce RBLS, isolates from infections with this variant have not replicated this phenotype (5, 8, 12). In this study, all G600D isolates were *emm*12 and isolated from Chicago, Portland, and Atlanta. Notably, no isolates with variant T553K were identified.

**Fig 1:**
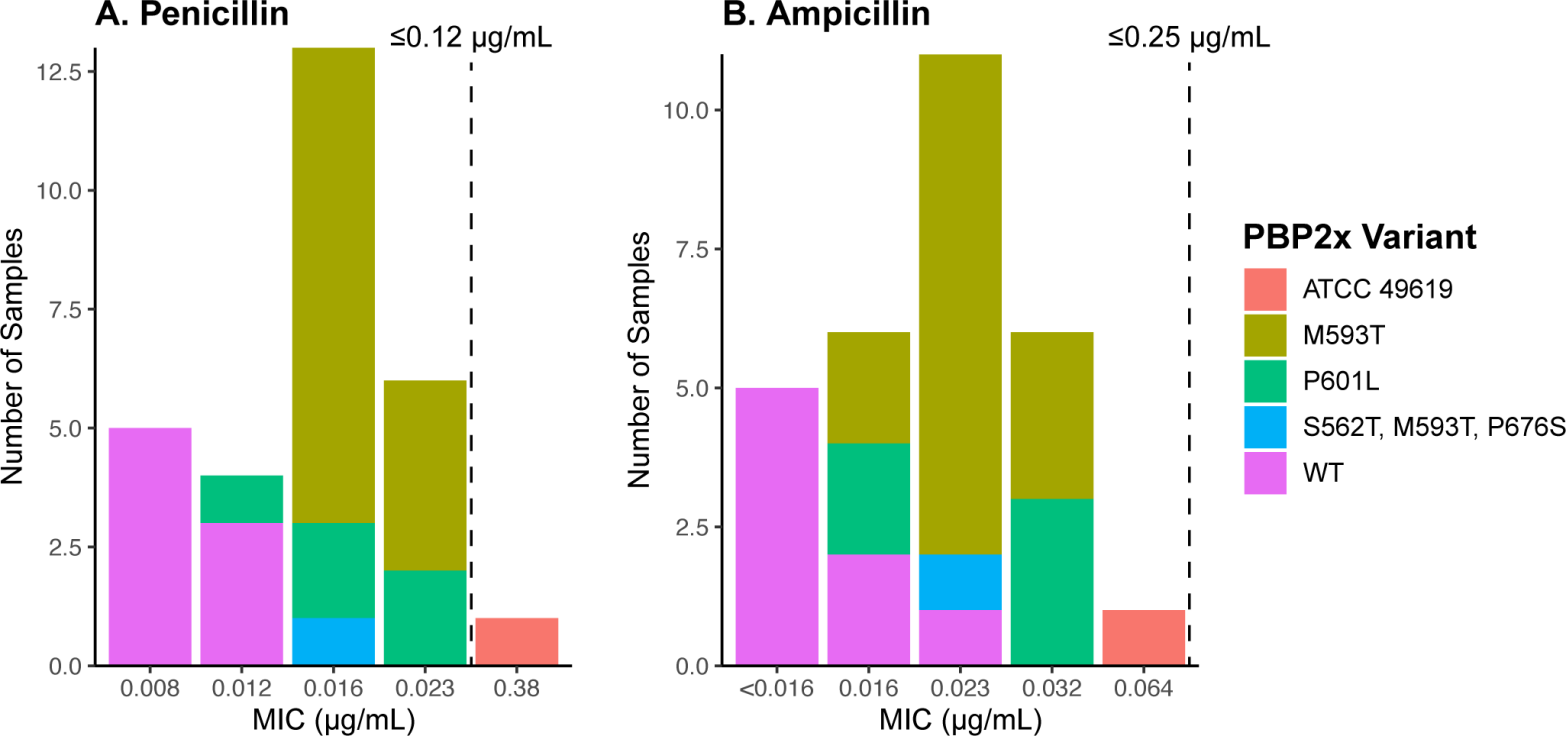
Minimum inhibitory concentrations of penicillin G (**A**) and ampicillin (**B**) were measured in *S. pyogenes* with PBP2x RBLS variants M593T (14); P601L (5); S562T, M593T, P676S (1); WT controls (8); and control strain *S. pneumoniae* ATCC 49619. Dashed vertical lines indicate the Clinical and Laboratory Standards Institute clinical resistance breakpoints for penicillin G and ampicillin (13).

The effect of PBP2x RBLS variants on penicillin and ampicillin susceptibility in pharyngitis isolates was evaluated through MIC testing using the agar gradient diffusion method (MTS, Liofilchem), following the manufacturer’s protocol with inoculum solution standardized to an optical density at 600 nm of ~0.4. Twenty isolates with PBP2x variants (14 M593T; 5 P601L; and 1 S562T, M593T, P676), 8 WT isolates, and control strain *Streptococcus pneumoniae* ATCC 49619 were tested in at least 2 biological replicates. Each replicate was independently interpreted by three investigators blinded to the PBP2x variants of the isolates, and the mode of the MICs was recorded. Final MIC values were determined as follows: the mode if at least two replicates were identical, the higher of the values if replicates differed by one dilution, and the median if three replicates were performed that each differed by one dilution. Compared to WT controls, isolates with PBP2x RBLS variants exhibited ~1.5–2× higher MICs to both penicillin and ampicillin. No differences in MICs between RBLS variants were observed (Fig. 1). Importantly, all isolates had MICs below the clinical resistance breakpoints (0.12 µg/mL for penicillin and 0.25 µg/mL for ampicillin) (13).

**Fig 2:**
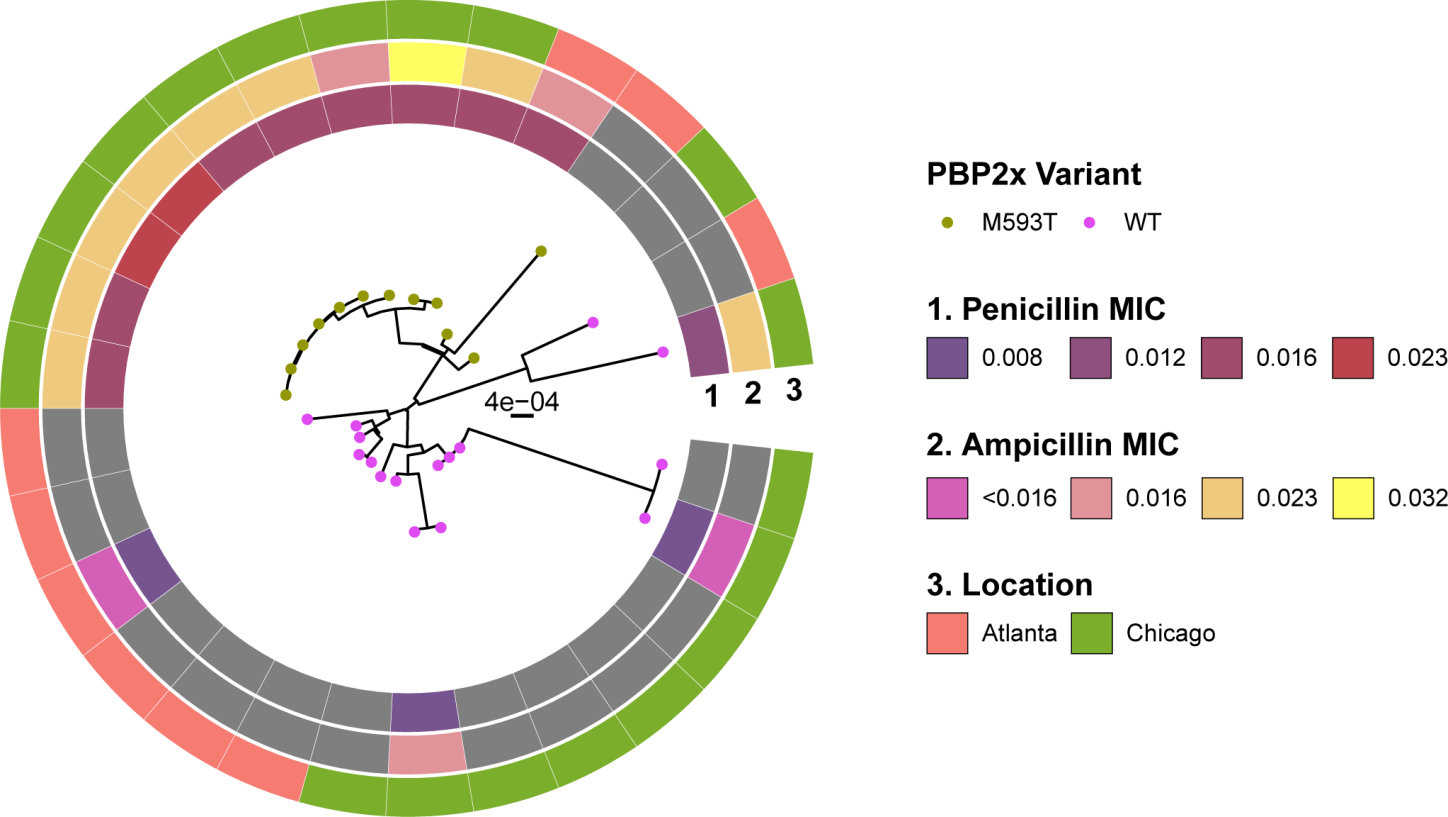
Midpoint-rooted maximum likelihood whole-genome phylogenetic tree of 28 GAS *emm* type *75* isolates from U.S. pediatric pharyngitis cases. Two genetic outlier specimens with WT PBP2x sequences were omitted to facilitate visualization of M593T specimens. Tip color represents the PBP2x variant, and rings represent MICs to penicillin (1) and ampicillin (2) and geographic origin of the isolate (3). Gray boxes indicate that susceptibility testing for the isolate was not performed. The scale bar represents 0.0004 substitutions per site. For the full tree with all 30 *emm*75 isolates, see Fig. S1.

As most isolates with PBP2x RBLS variants were *emm75* and carried M593T, we further examined the phylogenetic relationships among the 30 *emm75* isolates collected in this study. WGS reads of PBP2x WT and RBLS allele-carrying *emm75* isolates were aligned to an *emm*75 reference sequence (RefSeq GCF_005163865.1) using the BWA-MEM algorithm (14). A maximum-likelihood phylogenetic tree based on variable positions from whole-genome consensus sequences produced by bcftools v1.20 was generated using IQ-TREE 2 with the ModelFinder algorithm and was visualized using R v4.5.0 and ggtree v3.16.3 (15–18). Isolates with PBP2x M593T formed a unique clade among *emm75* isolates, averaging 62.0 ± 64.6 pairwise nucleotide differences with two sets of highly clonal isolates differing by 0–4 pairwise single nucleotide variants (Fig. 2). These results are consistent with the phylogenetic clustering of U.S. invasive *emm75*/M593T specimens identified by Chochua et al. (4) and an expanded analysis of globally distributed *emm75* isolates (Fig. S2).

The finding that rates of RBLS-associated PBP2x variant carriage among GAS pharyngitis isolates approximate those reported in invasive GAS is concerning and carries considerable public health implications. In the context of a potential survival advantage conferred by low-level β-lactam nonsusceptibility, these findings reinforce the importance of both antimicrobial stewardship and genomic surveillance of GAS infections across anatomic sites.

## Data Availability

Whole-genome sequencing assemblies and reads of all isolates were deposited to GenBank and the Sequencing Read Archive under accession PRJNA817826. Isolates with PBP2x RBLS and *emm*75 are highlighted in Table S2.

## SUPPLEMENTAL MATERIAL

Supplemental material aac.00354-26-s0001.docx Tables S1 and S2; Fig. S1 and S2.

ASM does not own the copyrights to Supplemental Material that may be linked to, or accessed through, an article. The authors have granted ASM a non-exclusive, world-wide license to publish the Supplemental Material files. Please contact the corresponding author directly for reuse.

## References

[1] Ebell MH, Smith MA, Barry HC, Ives K, Carey M. 2000. Does this patient have strep throat? JAMA 284:2912. doi:10.1001/jama.284.22.2912 11147989

[2] Committee on Infectious Diseases, American Academy of Pediatrics. 2024. Group A streptococcal infections. *In* Kimberlin DW, Banerjee R, Barnett ED, Lynfield R, Sawyer MH (ed), Red book: 2024–2027 report of the committee on infectious diseases. American Academy of Pediatrics, Itaska, IL.

[3] Vannice KS, Ricaldi J, Nanduri S, Fang FC, Lynch JB, Bryson-Cahn C, Wright T, Duchin J, Kay M, Chochua S, Van Beneden CA, Beall B. 2020. Streptococcus pyogenes pbp2x mutation confers reduced susceptibility to β-lactam antibiotics. Clin Infect Dis 71:201–204. doi:10.1093/cid/ciz1000 31630171 PMC7167332

[4] Chochua S, Metcalf B, Li Z, Mathis S, Tran T, Rivers J, Fleming-Dutra KE, Li Y, McGee L, Beall B. 2022. Invasive group A streptococcal penicillin binding protein 2× variants associated with reduced susceptibility to β-lactam antibiotics in the United States, 2015–2021. Antimicrob Agents Chemother 66:e0080222. doi:10.1128/aac.00802-22 35969070 PMC9487518

[5] Musser JM, Beres SB, Zhu L, Olsen RJ, Vuopio J, Hyyryläinen HL, Gröndahl-Yli-Hannuksela K, Kristinsson KG, Darenberg J, Henriques-Normark B, Hoffmann S, Caugant DA, Smith AJ, Lindsay DSJ, Boragine DM, Palzkill T. 2020. Reduced in vitro susceptibility of Streptococcus pyogenes to β-lactam antibiotics associated with mutations in the pbp2x gene is geographically widespread. J Clin Microbiol 58:e01993-19. doi:10.1128/JCM.01993-19 31996443 PMC7098749

[6] Southon SB, Beres SB, Kachroo P, Saavedra MO, Erlendsdóttir H, Haraldsson G, Yerramilli P, Pruitt L, Zhu L, Musser JM, Kristinsson KG. 2020. Population genomic molecular epidemiological study of macrolide-resistant Streptococcus pyogenes in Iceland, 1995 to 2016: identification of a large clonal population with a pbp2x mutation conferring reduced in vitro β-lactam susceptibility. J Clin Microbiol 58:e00638-20. doi:10.1128/JCM.00638-20 32522827 PMC7448646

[7] Hayes A, Lacey JA, Morris JM, Davies MR, Tong SYC. 2020. Restricted sequence variation in Streptococcus pyogenes penicillin binding proteins. mSphere 5:e00090-20. doi:10.1128/mSphere.00090-20 32350098 PMC7193039

[8] Beres SB, Zhu L, Pruitt L, Olsen RJ, Faili A, Kayal S, Musser JM. 2022. Integrative reverse genetic analysis identifies polymorphisms contributing to decreased antimicrobial agent susceptibility in Streptococcus pyogenes. mBio 13:e0361821. doi:10.1128/mbio.03618-21 35038921 PMC8764543

[9] Chen S, Zhou Y, Chen Y, Gu J. 2018. fastp: an ultra-fast all-in-one FASTQ preprocessor. Bioinformatics 34:i884–i890. doi:10.1093/bioinformatics/bty560 30423086 PMC6129281

[10] Prjibelski A, Antipov D, Meleshko D, Lapidus A, Korobeynikov A. 2020. Using SPAdes de novo assembler. Curr Protoc Bioinformatics 70:e102. doi:10.1002/cpbi.102 32559359

[11] Parks DH, Imelfort M, Skennerton CT, Hugenholtz P, Tyson GW. 2015. CheckM: assessing the quality of microbial genomes recovered from isolates, single cells, and metagenomes. Genome Res 25:1043–1055. doi:10.1101/gr.186072.114 25977477 PMC4484387

[12] Chochua S, Metcalf BJ, Li Z, Rivers J, Mathis S, Jackson D, Gertz RE Jr, Srinivasan V, Lynfield R, Van Beneden C, McGee L, Beall B. 2017. Population and whole genome sequence based characterization of invasive group a streptococci recovered in the United States during 2015. mBio 8:e01422-17. doi:10.1128/mBio.01422-17 28928212 PMC5605940

[13] CLSI. 2025. CLSI supplement M100. In Performance standards for antimicrobial susceptibility testing, 35th ed. Clinical and Laboratory Standards Institute.

[14] Li H. 2013. Aligning sequence reads, clone sequences and assembly contigs with BWA-MEM. arXiv. doi:10.48550/arXiv.1303.3997

[15] Danecek P, Bonfield JK, Liddle J, Marshall J, Ohan V, Pollard MO, Whitwham A, Keane T, McCarthy SA, Davies RM, Li H. 2021. Twelve years of SAMtools and BCFtools. Gigascience 10:giab008. doi:10.1093/gigascience/giab008 33590861 PMC7931819

[16] Minh BQ, Schmidt HA, Chernomor O, Schrempf D, Woodhams MD, von Haeseler A, Lanfear R. 2020. IQ-TREE 2: new models and efficient methods for phylogenetic inference in the genomic era. Mol Biol Evol 37:1530–1534. doi:10.1093/molbev/msaa015 32011700 PMC7182206

[17] R Core Team. 2025. R: a language and environment for statistical computing. https://www.R-project.org.

[18] Yu G, Smith DK, Zhu H, Guan Y, Lam TT. 2017. ggtree: an r package for visualization and annotation of phylogenetic trees with their covariates and other associated data. Methods Ecol Evol 8:28. doi:10.1111/2041-210X.12628

