# Supplementary material for: Identification of Streptococcus pyogenes isolates with reduced beta-lactam susceptibility in a cohort of children with pharyngitis
Source: Antimicrob Agents Chemother. 2026 May 29;70(7):e00354-26. doi: 10.1128/aac.00354-26 (PMC13321798; doi:10.1128/aac.00354-26)
Supplement: Supplemental material — Tables S1 and S2; Fig. S1 and S2. [file aac.00354-26-s0001.docx]

**Tables**

**Table S1 Penicillin-binding protein 2x (PBP2x) variants identified**
10 unique PBP2x variants were identified across 902 samples. Variants previously associated with reduced β-lactam susceptibility by Chochua *et al.* (2022) accounted for 2.8% of samples and are shown in bold.

| **PBP2x ID** | **Amino Acid Variant** | **Count (Percent)** |
| --- | --- | --- |
| 1 | WT | 662 (73.4%) |
| 4 | G600D | 108 (12.0%) |
| 3 | S562T | 58 (6.4%) |
| 8 | I502V, P676S | 46 (5.1%) |
| **10** | **M593T** | **17 (1.9%)** |
| **7** | **P601L** | **6 (0.7%)** |
| 73 | V547I | 2 (0.2%) |
| 2 | D353A, P676S | 1 (0.1%) |
| **20** | **P601H** | **1 (0.1%)** |
| **46** | S562T, **M593T**, P676S | **1 (0.1%)** |

**Table S2 PBP2x RBLS and *emm*75 isolates**

Whole-genome sequence assemblies and reads for all analyzed isolates were deposited to NCBI under BioProject PRJNA817826. Those with *emm* type 75 or PBP2x variants previously associated with reduced β-lactam susceptibility (RBLS) by Chochua *et al.* (2022) are listed.

| **Sample Name** | ***emm* Type** | **PBP2x ID** | **Biosample Accession Number** |
| --- | --- | --- | --- |
| GAS-115827 | 75 | 10 | SAMN52394252 |
| GAS-126565 | 75 | 10 | SAMN52394241 |
| GAS-161448 | 75 | 1 | SAMN52394225 |
| GAS-191193 | 4 | 10 | SAMN52394264 |
| GAS-219690 | 75 | 1 | SAMN52394227 |
| GAS-233953 | 4 | 10 | SAMN52394260 |
| GAS-235821 | 75 | 10 | SAMN52394253 |
| GAS-243707 | 75 | 1 | SAMN52394232 |
| GAS-274360 | 75 | 1 | SAMN52394226 |
| GAS-308908 | 1 | 7 | SAMN52394254 |
| GAS-312618 | 75 | 1 | SAMN52394229 |
| GAS-322247 | 75 | 1 | SAMN52394228 |
| GAS-324070 | 4 | 10 | SAMN52394262 |
| GAS-368277 | 1 | 7 | SAMN52394255 |
| GAS-381019 | 75 | 1 | SAMN52394239 |
| GAS-464941 | 75 | 1 | SAMN52394231 |
| GAS-513762 | 75 | 1 | SAMN52394233 |
| GAS-519161 | 1 | 7 | SAMN52394256 |
| GAS-530230 | 75 | 10 | SAMN52394236 |
| GAS-530423 | 75 | 10 | SAMN52394237 |
| GAS-532479 | 75 | 1 | SAMN52394246 |
| GAS-553440 | 75 | 1 | SAMN52394247 |
| GAS-567855 | 75 | 1 | SAMN52394245 |
| GAS-572341 | 75 | 1 | SAMN52394251 |
| GAS-608064 | 75 | 10 | SAMN52394238 |
| GAS-615899 | 11 | 20 | SAMN52394259 |
| GAS-650513 | 4 | 10 | SAMN52394263 |
| GAS-666442 | 4 | 10 | SAMN52394261 |
| GAS-680279 | 75 | 1 | SAMN52394249 |
| GAS-686793 | 75 | 1 | SAMN52394242 |
| GAS-716407 | 75 | 10 | SAMN52394243 |
| GAS-769122 | 75 | 1 | SAMN52394250 |
| GAS-758820 | 75 | 10 | SAMN53033535 |
| GAS-790155 | 87 | 7 | SAMN52394266 |
| GAS-807712 | 1 | 7 | SAMN52394257 |
| GAS-862079 | 75 | 10 | SAMN52394244 |
| GAS-882353 | 75 | 1 | SAMN52394230 |
| GAS-903448 | 75 | 10 | SAMN52394240 |
| GAS-923678 | 75 | 10 | SAMN52394235 |
| GAS-934231 | 11 | 7 | SAMN52394258 |
| GAS-955771 | 75 | 10 | SAMN52394234 |
| GAS-976345 | 75 | 1 | SAMN52394248 |
| GAS-986663 | 73 | 46 | SAMN52394265 |

**Figures**

**Fig. S1** Midpoint rooted maximum likelihood whole-genome phylogenetic tree of all 30 GAS *emm* type *75* isolates from US pediatric pharyngitis cases. Tip color represents the PBP2x variant. The scale bar represents 0.008 substitutions per site.

**Fig. S2** Midpoint rooted maximum likelihood 95% core genome phylogenetic tree of 759 globally distributed *emm75* GAS isolates. The 30 *emm75* isolates from this study were pooled with an additional 729 paired-end Illumina sequences obtained from NCBI. Tip color represents the *emm* subtype, and rings represent the PBP2x variant (1), country of isolation (2), and source of the sequence (3). The scale bar represents 0.01 substitutions per site. All non-WT PBP2x variants were previously associated with RBLS. Of these, all but 1 isolate (Australia) were sampled from the USA.

**
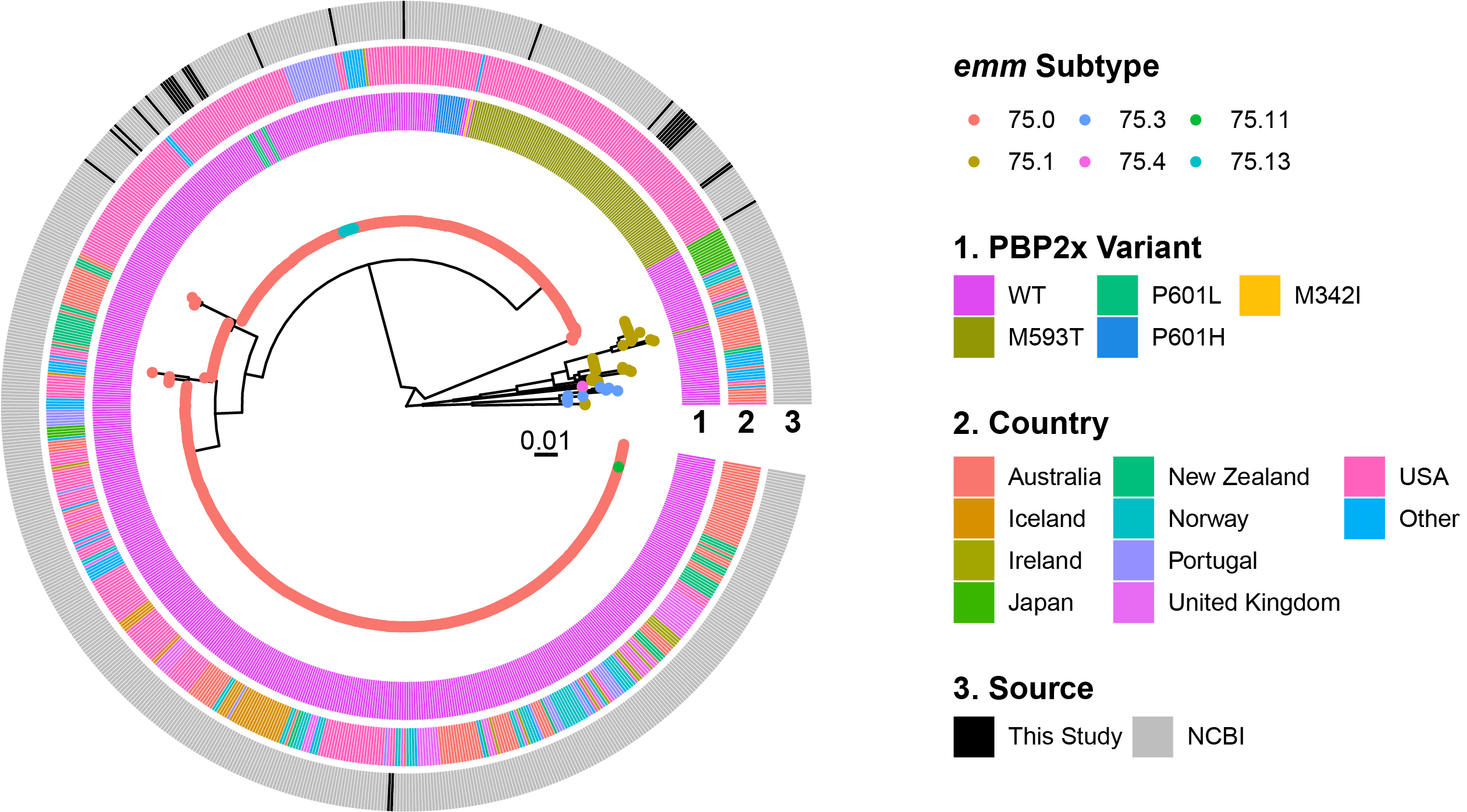
**
